# Bedside Ultrasound for Ventricular Size Monitoring in Patients with PEEK Cranioplasty: A Preliminary Experience of Technical Feasibility in Neurotrauma Setting

**DOI:** 10.1007/s12028-022-01544-w

**Published:** 2022-06-27

**Authors:** Francesco Signorelli, Giuseppe Maria Della Pepa, Giammaria Marziali, Eleonora Ioannoni, Alessandro Olivi, Anselmo Caricato, Massimiliano Visocchi, Nicola Montano

**Affiliations:** 1 Department of Neurosurgery, Fondazione Policlinico Universitario Agostino Gemelli Istituto di Ricovero e Cura a Carattere Scientifico, Rome, Italy; 2 Department of Radiology, Fondazione Policlinico Universitario Agostino Gemelli Istituto di Ricovero e Cura a Carattere Scientifico, Rome, Italy; 3 Department of Anesthesiology and Intensive Care Medicine, Fondazione Policlinico Universitario Agostino Gemelli Istituto di Ricovero e Cura a Carattere Scientifico, Rome, Italy; 4 Department of Neuroscience, Neurosurgery Section, Fondazione Policlinico Universitario Agostino Gemelli Istituto di Ricovero e Cura a Carattere Scientifico, Rome, Italy

**Keywords:** Cranioplasty, Brain echography, Shunt, Hydrocepalus, PEEK, TBI, Transcranial ultrasound

## Abstract

**Background:**

Posttraumatic hydrocephalus is a known complication after traumatic brain injury, particularly affecting patients undergoing decompressive craniectomy. Posttraumatic hydrocephalus monitoring in these patients represents a common issue in neurosurgical practice. Patients require periodical assessments by means of computed tomography (CT) scans. This study presents a preliminary institutional series in which ultrasound was used as a bedside imaging technique to monitor ventricular size in patients harboring a polyetheretherketone (PEEK) cranioplasty. Exploiting the PEEK cranioplasty permeability to echoes, we evaluated the feasibility of this bedside imaging method in monitoring hydrocephalus evolution, determining effects of ventriculo-peritoneal shunt, and excluding complications.

**Methods:**

Eight patients with traumatic brain injury harboring PEEK cranioplasty following decompressive craniectomy were prospectively evaluated. Ultrasound measurements were compared with CT scan data taken the same day, and ventricular morphometry parameters were compared.

**Results:**

Ultrasound images through the PEEK cranioplasty were of high quality and intracranial anatomy was distinctly evaluated. A strong correlation was observed between ultrasound and CT measurements. Concerning distance between lateral ventricles frontal horns (IFH) and the diameter of the third ventricle (TV), we found a strong correlation between transcranial sonography and CT measurements in preventriculoperitoneal shunt (rho = 0.92 and *p* = 0.01 for IFH; rho = 0.99 and *p* = 0.008 for TV) and in postventriculoperitoneal shunt examinations (rho = 0.95 and *p* = 0.03 for IFH; rho = 0.97 and *p* = 0.03 for TV). The mean error rate between transcranial sonography and CT scan was 1.77 ± 0.91 mm for preoperative IFH, 0.65 ± 0.27 mm for preoperative TV, 2.18 ± 0.82 mm for postoperative IFH, and 0.48 ± 0.21 mm for postoperative TV.

**Conclusions:**

Transcranial ultrasound could represent a simplification of the follow-up and management of ventricular size of patients undergoing PEEK cranioplasty. Even if this is a small series, our preliminary results could widen the potential benefits of PEEK, not only as effective material for cranial reconstruction but also, in selected clinical conditions, as a reliable window to explore intracranial content and to monitor ventricular sizes and shunt functioning.

## Introduction

Surgical restoration of the integrity of cranial vault by means of cranioplasty after decompressive craniectomy (DC) for traumatic brain injury (TBI) provides a combination of cerebral protection and cosmetic improvement. In some cases, it reestablishes the regular distribution of cerebrospinal fluid (CSF) along the cortical surface, thereby restoring physiological CSF circulation and absorption. Nonetheless, in some other cases, posttraumatic hydrocephalus (PTH) may occur, particularly in patients who underwent DC [1, 2], with a reported incidence in the literature ranging widely from 7.9 to 27% [1–3]. Recent studies on CSF hydrodynamics demonstrated the role of penetrating arterial pulsatility within the cerebral cortex in generating a hydrostatic force that drives CSF flow into the parenchyma and along perivascular spaces and interstitial fluid clearance from the brain’s extracellular space (glymphatic system) [4, 5]. The opening of intracranial compartment to atmospheric pressure after decompressive craniectomy results in reduced arterial pulsatility, decreased cerebral blood flow, and impaired brain glymphatic clearance, which overall contribute to formations of delayed abnormal collections of fluid, including ipsilateral, parafalcine or contralateral hygromas, or ventriculomegaly. Parafalcine hygromas often precede hydrocephalus after TBI, but it can arise independently from hygromas [4, 5]. PTH itself is associated with a poor neurological outcome following TBI [6]. If not promptly recognized, it can lead to brain metabolism disturbance with further neurological compromise and impaired recovery [1–3, 6]. With few exceptions, the placement a of ventriculoperitoneal shunt (VPS) remains the first-choice treatment, with early shunting providing favorable outcomes during rehabilitation [7]. After TBI, PTH may appear as a late complication, especially in the presence of intraventricular hemorrhage or subdural hygroma [8]. Hence, in selected cases, the risk of developing PTH needs to be periodically assessed by means of computed tomography (CT) or magnetic resonance imaging (MRI) scans [9, 10].

Given the lower rates of infection, low cost of conservation, and assured biocompatibility, the autologous cranial flap remains the gold standard for cranial reconstruction. Nevertheless, an alloplastic prosthesis is preferred in cases of comminuted fractures, penetrating trauma, and bone flap infection or resorption. Several materials, including metals, acrylics, ceramics, and plastics, have been used, with varying degrees of success in using titanium mesh, polymethyl methacrylate, and polyetheretherketone (PEEK), the most commonly used materials [9–11].

PEEK is a chemically inert compound, sharing with cortical bone analogous tensile properties and similar modulus of elasticity [12]. PEEK implants offer several advantages compared with other materials, including strength, stiffness, durability, and inertness. Moreover, it has a low thermal conductivity unlike metallic implants and has a natural radiolucency, which makes it compatible with ultrasound and MRI. Hence, it is nowadays one of the most common options in posttraumatic cranial vault reconstruction.

Transcranial sonography (TCS) is a feasible, safe, noninvasive, and low-cost imaging technique that has been recently introduced in several neurosurgical settings both intraoperatively and bedside [13–15]. However, its application in neurosurgery has been generally limited to the intraoperative time, after bone flap is removed, because ultrasound beams cannot penetrate bone as it casts an acoustic shadow beyond it. Apart from intraoperative applications, TCS is generally limited by skull bone but can be exploited where the skull is thin, such as the temporal bone, providing only limited morphological information [16]. Conversely, in decompressed patients, TCS has already demonstrated feasible and reliable in identifying a series of complications using the craniectomy skin flap as an acoustic window, avoiding the need of more demanding examinations such as CT or MRI [17].

However, studies in the literature on real-time TCS monitoring through a cranioplasty window are limited, and only a few experiences have been published with a specific focus on PTH and PEEK sonolucent cranioplasty [18, 19].

The aim of the present study is to report our preliminary experience with TCS in evaluating ventricular size in patients with TBI harboring a PEEK cranioplasty. Moreover, we assessed the reliability of the echogenicity provided by this material in this definite setting.

## Materials and Methods

We prospectively evaluated the clinical and radiological data of patients with TBI harboring PEEK cranioplasty following DC and needing ventricular size monitoring between January 2019 and September 2021 at the Department of Neurosurgery, Fondazione Policlinico Universitario Agostino Gemelli Istituto di Ricovero e Cura a Carattere Scientifico, Catholic University, in Rome, Italy.

Overall, our institution admits more than 400 patients with TBI yearly, approximately 40 of whom undergo decompressive craniectomy. Notwithstanding autologous bone flap repositioning remains the gold standard for the patients who survive, around 15 cases per year receive PEEK cranioplasty.

Patients underwent PEEK cranioplasty and simultaneous or subsequent VPS implantation or came to our attention from the rehabilitation center for a worsening of neurological performances suggestive of the development of PTH.

The manufacturing of the implant had been preceded by preoperative radiologic studies by means of acquisition of a thin-cut skull CT scan with preset parameters in order to achieve excellent reconstructive results: matrix 512 × 512, slice thickness 1.0 mm, feed per rotation 1.0 mm, reconstructed slice increment 1.0 mm, reconstructed algorithm bone, and gantry tilt 0. CT data sets were shared and sent in Digital Imaging and Communications in Medicine format to the Manufacturer Firm (Synthes).

Ultrasound scans were performed by the senior Neurointensivist, expert in TCS (A.C.), who was blinded to the CT measurements. TCS examination was performed at patient bedside.

The ultrasound system was Toshiba Xario 200 with convex probe directly placed over patient skin overlying the cranioplasty.

For each patient, we used linear and curved probes. During the examination, the patient was in a supine position and the operator at the head of the table at the side of cranioplasty. The probe was positioned onto the skin at the flattest area of the bone flap to exploit the best window possible and then moved appropriately in order to achieve the axial projection that allowed the best visualization of the ventricular system. When visualization was optimal, the operator carried out the measurements. Time required to perform a TCS was around 20 min.

As the aim of our study was to explore reliability of the sonic window provided by PEEK cranioplasty, we compared standard CT scan images with ultrasounds images performed on the same day of CT scan (the day before the VPS placement and two days after the operation). Accordingly, we collected both quantitative and qualitative data. For the former, we measured parameters typical for ventricular morphometry such as maximum distance between lateral ventricles frontal horns (IFH) and diameter of the third ventricle (TV). The third ventricle has been identified as an anechogenic area bordered by hyperechogenic lines where the ultrasound beams meet the ependyme in an orthogonal manner. The diameter was assessed by measuring the distance between the inner boundaries of both hyperechogenic lines. The connecting distance-line had to be absolutely vertical. Same measurements were calculated on axial CT scan. Then, we compared the results by calculating pre and postoperative IFH and TV ratios between TCS and CT scan values (TCS/CT).

For qualitative data, we considered identification on TCS of falx, choroid plexus, the ventricular catether and hemoventricle.

The correlation between TCS and TC values were evaluated using the Spearman’s rank correlation test. A *p* value < 0.05 was considered significant.

Ethical approval was waived by the local committee due to the anonymous collection of clinical data obtained without invasive maneuvers and according to normal clinical practice.

## Results

This study included eight consecutive patients. Clinical and demographic data of patients are summarized in Table 1. Five patients underwent a right fronto-temporal-parietal cranioplasty and three patients underwent a left fronto-temporal-parietal cranioplasty. The main reason for a PEEK cranioplasty procedure was the infection of the autologous bone flap (four patients); in two patients, the spontaneous resorption of the autologous bone flap was the reason, and in the remaining two patients, the presence of multiple fragments was the reason for undergoing PEEK cranioplasty. Six patients developed PTH and required a VPS (a Codman Hakim programmable valve was used in all cases). Two patients had moderate ventricular enlargement without developing clinical and radiological signs of hydrocephalus. The shape and size of cranioplasty varied, and the thickness ranged from 6.0 to 6.8 mm.

**Table 1:** Patients’ characteristics

Patient number	Sex	Age (yr)	CP indication	Flap site (side)	Shunt (Y/N)	Mean flap thickness (mm)
1	M	18	Infection	FTP (left)	Y	6.2
2	M	24	Resorption	FTP (right)	Y	6.4
3	M	27	Comminuted fracture	FTP (right)	Y	6.6
4	F	35	Infection	FTP (right)	Y	6.1
5	M	29	Comminuted fracture	FTP (right)	N	6.0
6	F	38	Resorption	FTP (left)	N	6.8
7	F	21	Infection	FTP (right)	Y	6.1
8	M	29	Infection	FTP (left)	Y	6.6

*CP* Cranioplasty, *F* Female, *FTP* Fronto-temporo-parietal, *M* Male, *N* No, *Y* Yes

**Fig. 1:**
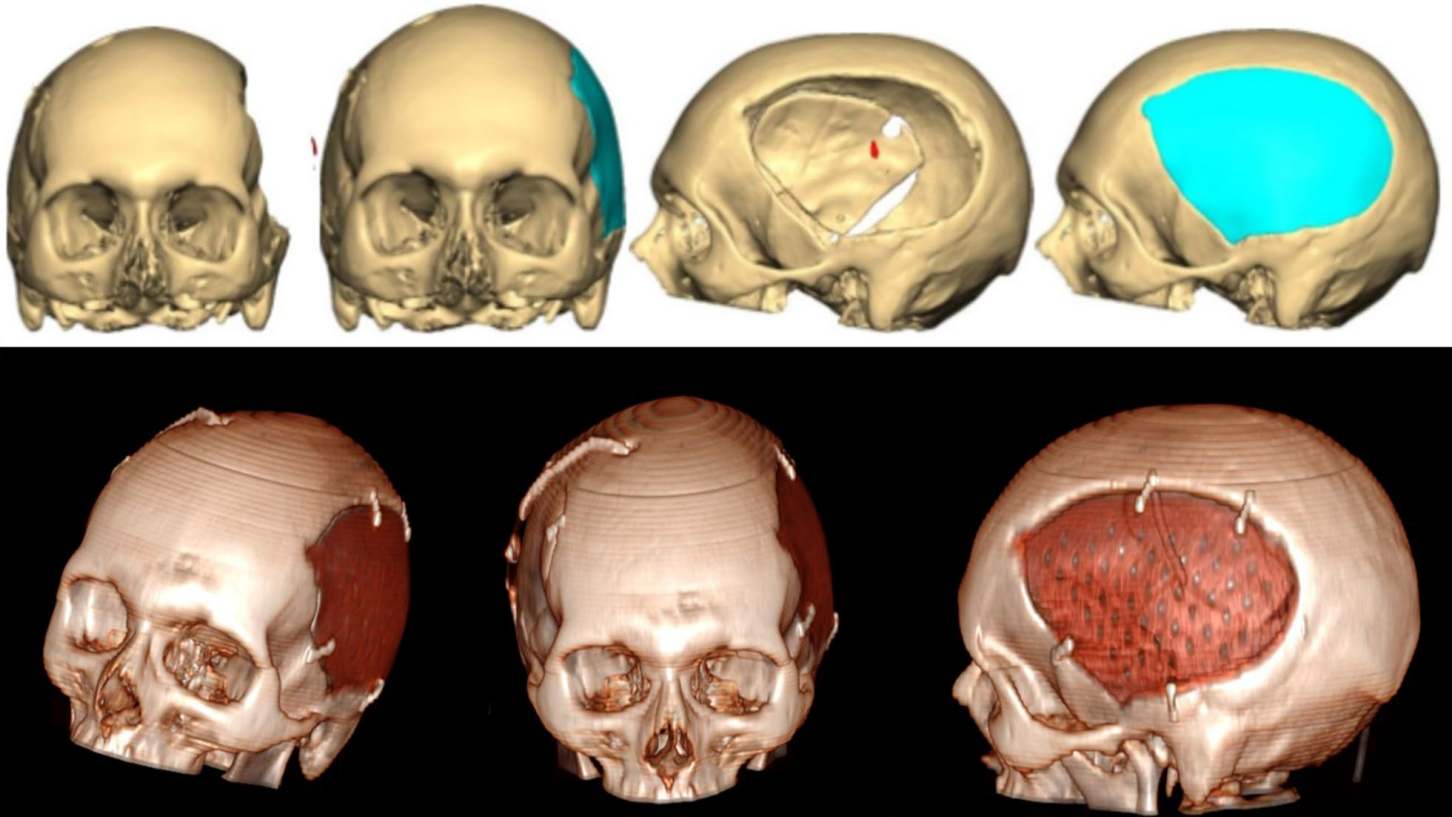
Tridimensional rendering of implanted PEEK cranioplasty. The relative “permeability” of PEEK to echoes allows for high quality intracranial ultrasound evaluation. PEEK, polyetheretherketone

Figure 1 shows tridimensional rendering of implanted PEEK cranioplasty.

Ultrasound frequency, focus, and imaging brightness were regulated according to the depth and kind of structure under evaluation. The best results in terms of visualization were obtained with 3-MHz to 9-MHz linear transducers. Lower frequency was used for deeper structures and a higher frequency was used for more superficial elements.

Concerning IFH and TV, we found a strong correlation between TCS and CT measurements, both in pre-VPS (rho = 0.92 and *p* = 0.01 for IFH; rho = 0.99 and *p* = 0.008 for TV) and in post-VPS examinations (rho = 0.95 and *p* = 0.03 for IFH; rho = 0.97 and *p* = 0.03 for TV), as summarized in Table 2.

**Table 2:** CT and TCS measurements

Patient number	Mode	Preoperative IFH (mm)	Preoperative TV (mm)	Postoperative IFH (mm)	Postoperative TV (mm)	Preoperative IFH ratio (TCS/CT)	Preoperative TV ratio (TCS/CT)	Postoperative IFH ratio (TCS/CT)	Postoperative TV ratio (TCS/CT)
1	TCS	65.7	22.2	54.7	16.6	0.96	1.01	0.93	0.99
	CT	68.2	21.8	58.3	16.8				
2	TCS	67.1	24.4	56.1	17.5	0.96	1.02	0.96	0.98
	CT	69.8	23.8	58.4	17.9				
3	TCS	59.2	19.3	52.2	15.5	1.04	1.03	1.04	1.05
	CT	56.4	18.6	50.3	14.8				
4	TCS	58.3	19.1	52.2	15.5	1.03	1.06	1.04	1.05
	CT	56.5	17.9	50.3	14.8				
5	TCS	60.2	23.1	52.2	16.5	1.02	1.03	0.98	0.98
	CT	58.8	22.3	53.3	16.8				
6	TCS	60.1	24.4	56.1	17.5	0.98	0.97	0.96	0.96
	CT	61.2	25.1	58.4	18.1				
7	TCS	51.2	16.2	–	–	0.99	0.98	–	–
	CT	51.4	16.5	–	–				
8	TCS	52.2	15.5	–	–	1.03	1.03	–	–
	CT	50.3	15.0	–	–				

*CT* Computed tomography, *IFH* Distance between lateral ventricles frontal horns, *TCS* Transcranial sonography, *TV* Diameter of third ventricle

The mean error rate between TCS and ICS was 1.77 ± 0.91 mm for preoperative IFH, 0.65 ± 0.27 mm for preoperative TV, 2.18 ± 0.82 mm for postoperative IFH, and 0.48 ± 0.21 mm for postoperative TV.

The greatest difference was found in patient 4, with preshunt TV TCS/CT ratio of 1.06. Overall, we found a slight major discrepancy in IFH measurements compared with TV. The flap curvature does not allow the probe to perfectly adhere to the skin and thus be perpendicular to the ventricular system. This could result in the loss of reliable measurements, especially concerning measurements of deeper structures such as TV. Therefore, our finding is to some extent unexpected; a possible explanation is that, concerning IFH, it is more difficult than TV to capture and measure comparable cuts between TCS and CT. Apart from the reliability of the measurements, the third ventricle was the most challenging parameter to evaluate, as the plane between the hyperechogenic ependymal line and the hypoechogenic thalamus was difficult to find. Furthermore, we cannot speculate about the role of cranioplasty thickness in influencing image quality, as we had a range of no more than 0.8 mm in our series.

All the main intracranial structures were satisfactorily visualized, and it was possible to perform the desired measurements in all patients.

**Fig. 2:**
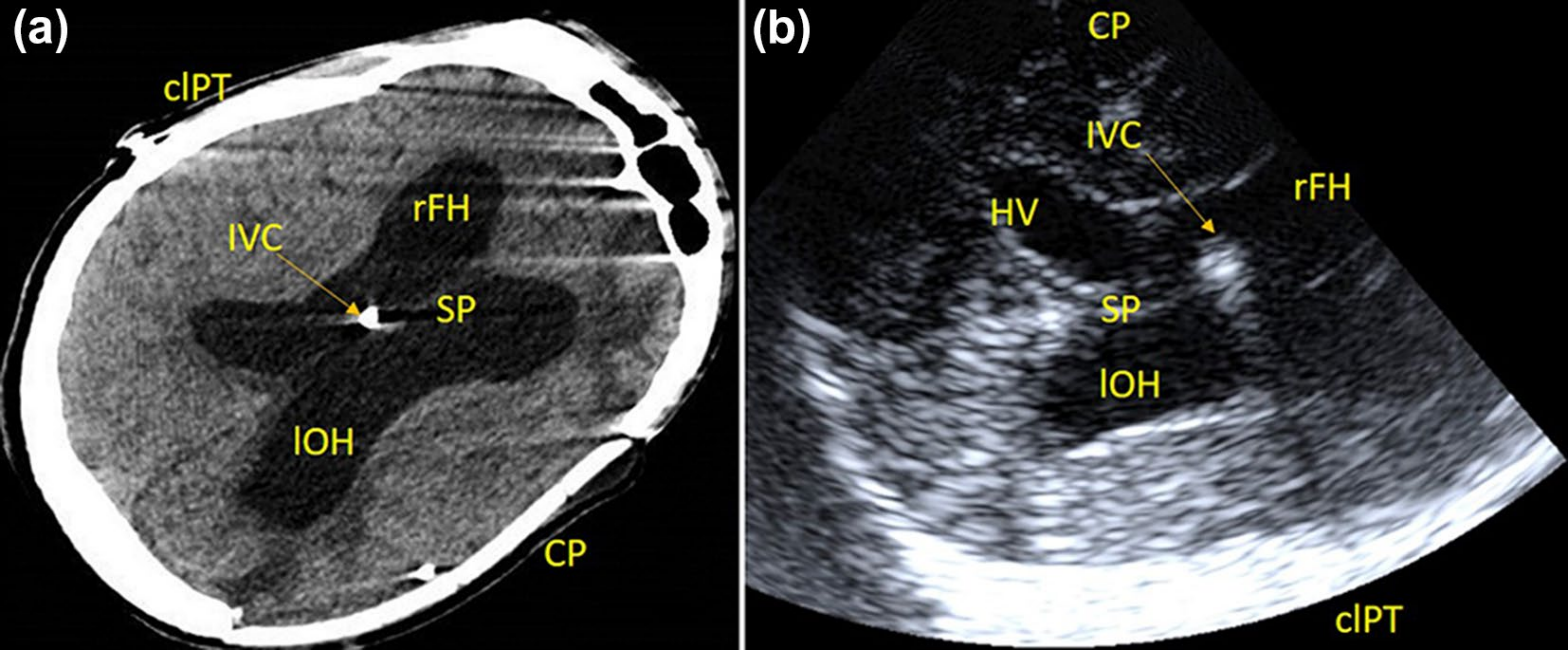
Intracranial evaluation before shunting (a, CT scan; b, transcranial ultrasound). Ventricular size and intracranial anatomy are clearly visible with transcranial ultrasound. In particular, the PEEK window allows us to also identify complications such as hemoventricle. clPT, contralateral parietal teca, CP, cranioplasty, CT, computed tomography, IVC, intraventricular catheter, lOH, left occipital horn, PEEK, polyetheretherketone, rFH, right frontal horn, SP, septum pellucidum

**Fig. 3:**
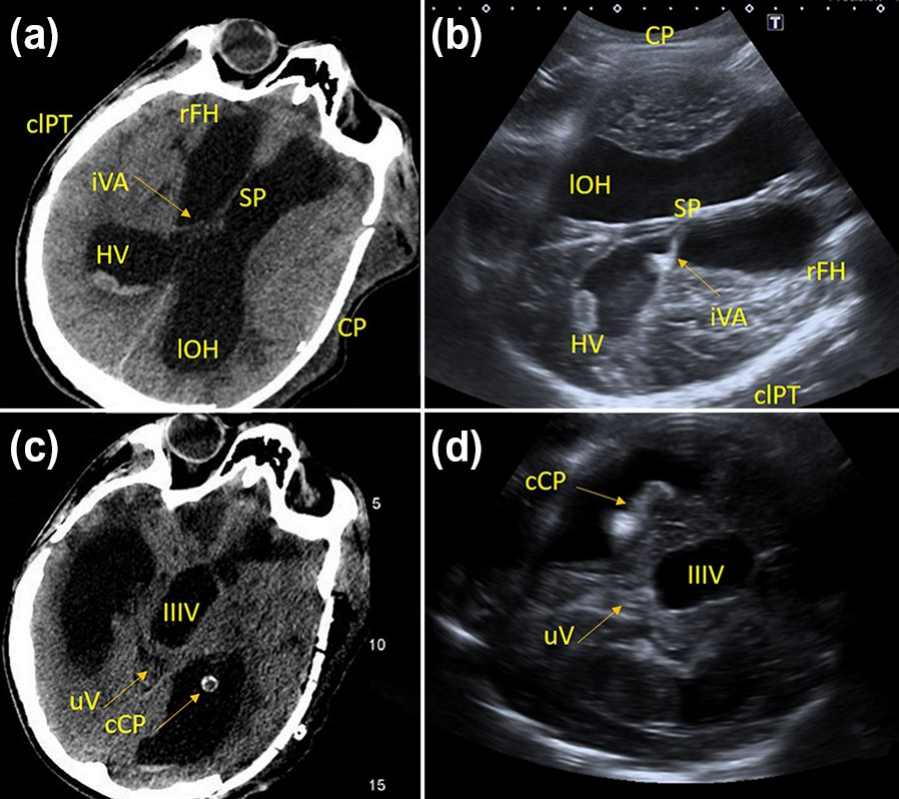
Postshunt evaluation at transcranial ultrasound (**b**, **c**) in comparison with CT (**a**, **d**). The course of catheter is clearly visible with transcranial ultrasound; ventricular size can be easily measured and monitored over time with bedside assessments. cCP, calcified choroid plexus, clPT, contralateral parietal teca, CP, cranioplasty, CT, computed tomography, HV, hemoventricle, IIIV, third ventricle, iVA, intraventricular adherences, lOH, left occipital horn, rFH, right frontal horn, SP, septum pellucidum, uV, upper vermis

**Fig. 4:**
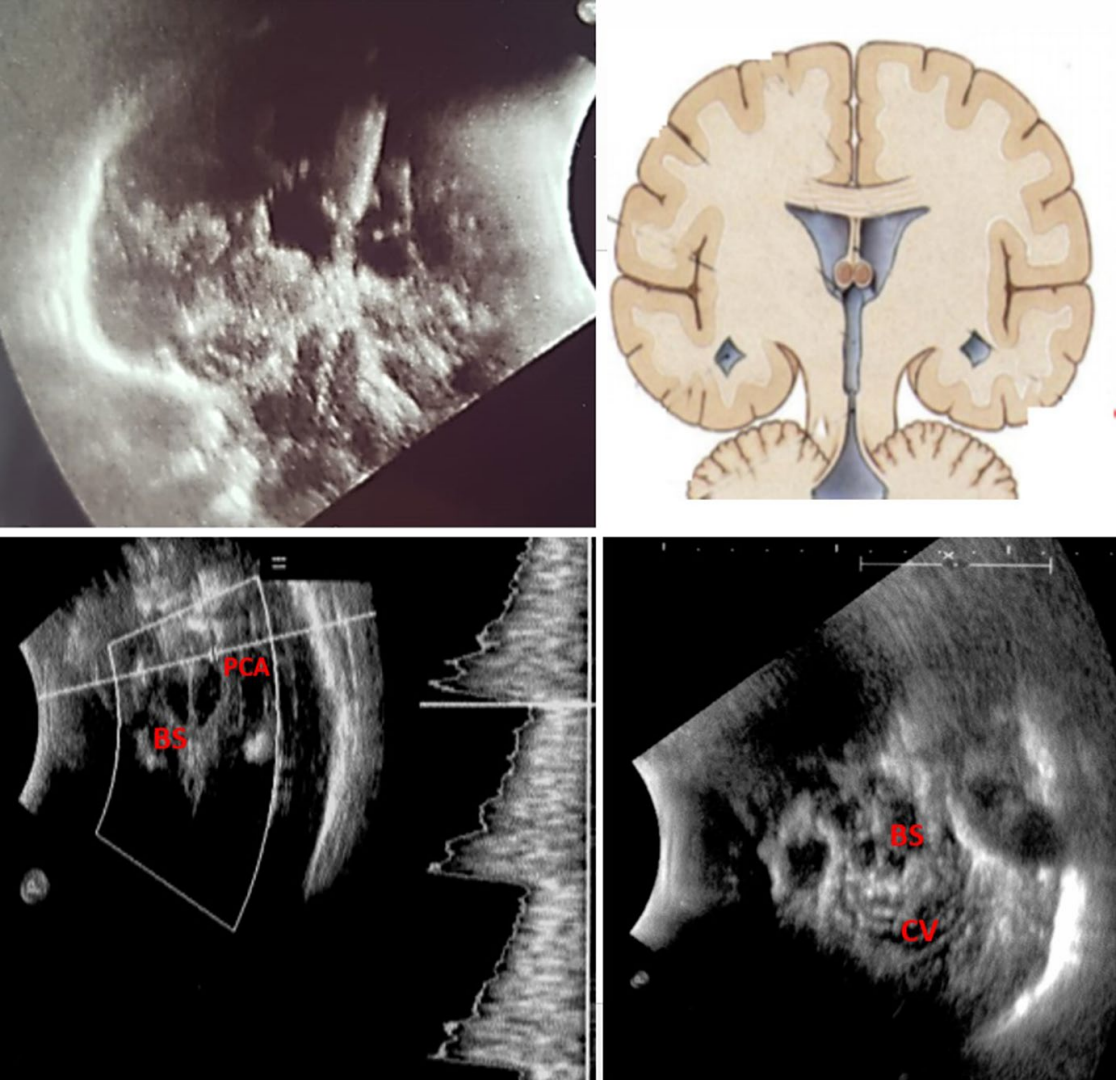
Exemplificative picture showing the possibility to study the whole intracranial anatomy through fronto-temporo-parietal PEEK cranioplasty. Upper row: the TCS probe is placed in a “coronal” projection; bran parenchyma, lateral ventricles, third ventricle, interhemispheric fissure, tentorium, mesencephalon and brainstem are clearly visible and easily recognizable (a similar anatomic projection in on the right as reference). Lower row: through the fronto-temporo-pariental window, posterior cranial fossa anatomy can be easily studied; Doppler and color-doppler tools can, indeed, be used to assess perfusion. BS, brainstem, CV, cerebellar vermis, PCA, posterior cerebral artery, PEEK, polyetheretherketone, TCS, transcranial sonography

As shown in Fig. 2, the presence of right-sided hemoventricle detected at CT was clearly visible at TCS in patient 1. Similarly, the course of catheter was also visible at TCS (Fig. 3). Among the other qualitative parameters, the falx and the choroidal plexus, posterior cranial fossa, and brainstem were clearly visible in all patients (Fig. 4).

**Fig. 5:**
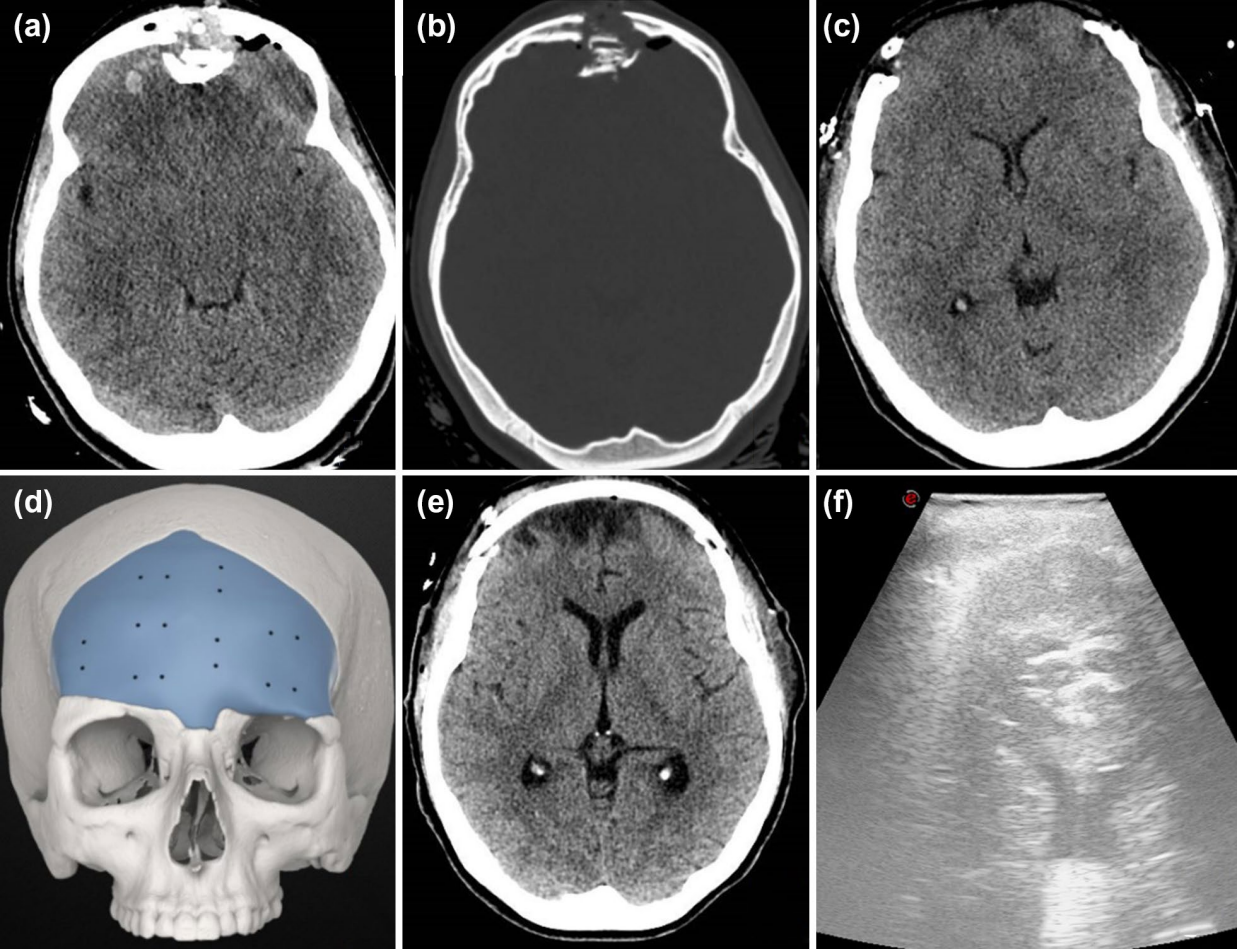
Preoperative CT scan showing anterior and posterior wall frontal sinus fracture along with bifrontal contusions (a, b) in a young patient who underwent bifrontal craniectomy with frontal sinus cranialization after TBI (c) and subsequent CP (d, e) in our hospital, not included in the present study. f, postcranioplasty ultrasound appearance of small ventricular size. CT, computed tomography, CP, cranioplasty, TBI, traumatic brain injury

Figure 5 shows TCS demonstrating small ventricular size in a young patient who underwent bifrontal craniectomy with frontal sinus cranialization after TBI and subsequent cranioplasty in our hospital; however, this patient was not included in the present study.

## Discussion

Although the relationship between DC and PTH still remains controversial, PTH is one of the most underrecognized and overwhelming complications after TBI in patients who undergo DC. Early detection and intervention in patients with TBI who develop PTH are of increasing clinical importance for preventing further neurologic compromises. Cranioplasty itself has proven to be helpful for the correction of the underlying CSF disturbances [20].

At our institution, after DC, we usually use PEEK cranioplasties as the first option in cases of comminuted fractures or in patients coming from other centers where they underwent their first surgery, and the appropriate cryoconservation is not guaranteed. Moreover, we use PEEK prosthesis in cases of bone flap infection or resorption [21].

Recently, De Bonis et al. [17] evaluated the feasibility of bedside evaluation in decompressed patients by means of serial TCS through the skin flap and observed an elevated diagnostic yield in identifying a series of conditions, including ischemic parenchymal areas, hydrocephalus, hygroma, brain herniation, basal cisterns patency cerebral contusions, epidural and subdural hematomas, and subarachnoid hemorrhage. The authors concluded that TCS could be a reliable alternative diagnostic tool for major complications in patients undergoing DC, thus limiting the number of CT scans per patient and overcoming several limitations such as costs, radiation exposure, and the need to move the patient [17]. As a matter of fact, like any soft tissue, craniectomy skin flap is an ideal acoustic window.

Recently, Mursch and Behnke-Mursch [22] illustrated preliminary results of TCS in two patients harboring PEEK cranioplasties. They demonstrated good visualization of intracranial structures and attributed this to the superior ultrasound permeability of PEEK compared with bone to the structural homogeneity of this synthetic material. On the other hand, other materials do not provide the same capability to be used as ultrasound windows to the intracranial space. Spena et al. [23] reported a nonoptimal quality of images in patients harboring polymethyl methacrylate flaps, which could potentially be overcome by manufacturing a dedicated concave probe. Confirming Mursh and and Behnke-Mursch’s experience, we did not experience such a problem with PEEK bone flaps.

On the heels of these seminal observations in our series, we investigated whether such information can be retrieved through a PEEK cranioplasty after cranial vault integrity has been reconstituted; indeed, we evaluated (1) whether transcranial ultrasound can be feasibly used *after* cranioplasty has been placed, and which anatomical details can be easily identified, and (2) whether this can possibly be considered an alternative to serial CT in monitoring the event and evolution of intracranial complications.

Our experience represents the first series of patients in whom an effective clinical use of TCS through PEEK cranioplasty has been performed. We were able to obtain very good quality images of the intracranial structures, optimal visualization of ventricular size and shape, and even the presence of hemoventricle in the left occipital horn, comparable with CT scan findings (as shown in Fig. 2). In our patients, we observed a strong correlation between ultrasound and CT data. It was possible to easily measure ventricular size to observe the general morphology of the ventricular system. In addition, the cranioplasty provided a good window to study the entire intracranial system, including pericerebral spaces, the posterior cranial fossa, the brainstem, and the mesencephalon (Fig. 4).

Moreover, through the PEEK cranioplasty window we were also able to assess the position of the ventricular catheter. We would stress the fact that, although TCS is an operator-dependent technique that requires a learning curve to provide reliable data, above all, such an evaluation is rapid, inexpensive, and can be repeated as many times as needed at the patient’s bedside; this possibly represents a simplified solution that eases the periodical evaluation of PTH and the effects of the shunt. Moreover, in emergency situations or in case of seizures or acute change of neurological status, suspected complications could be rapidly ruled out before performing CT.

Apart from the low number of patients and the drawbacks resulting from an operator-dependent technique, our study has several other limitations. First, the scarce adherence between the linear probe and the curvature of the flap can affect the accuracy of measurements, and this can represent a concern particularly when extreme precision is required. For example, sometimes shunt malfunction may be associated with only a slight increase of ventricular dimensions; in those cases, a difference of a few millimeters can be important and can guide management. Moreover, ultrasound imaging is widely available in neurotrauma centers and intensive care units, but the same cannot be said for rehabilitation centers. The onset of PTH is sometimes a late complication after cranioplasty in patients with TBI, occurring when he or she has already left the hospital; therefore, in those cases, directing the patient toward a radiological center to undergo CT could be easier than having a specialist expert in the field of cranial ultrasound available.

Although limited by the restricted number of patients, our current experience further strengthens the potential benefit of using PEEK not only as an effective material for cranial reconstruction [21] but also, in selected clinical conditions such as PTH, as a reliable window to explore intracranial content and to monitor ventricular sizes and shunt functioning.

Despite the aforementioned limitations, our encouraging preliminary results can be considered “a proof of concept” and drive future studies with the aim to explore further possible diagnostic implications offered by the technique of visualizing, for example, the whole brain parenchima, intracranial lesions, vascular malformations, and hemodynamic variations with Doppler TCS.

## Conclusions

Transcranial sonography is a reliable imaging technique for monitoring ventricular size in patients with TBI harboring or at risk of developing PTH through undergoing PEEK cranioplasty. Even though this is a small series consisting of eight cases, our preliminary results could widen the potential benefits of PEEK, not only as an effective material for cranial reconstruction but also, in selected clinical conditions, as a reliable window to explore intracranial content and to monitor ventricular sizes and shunt functioning.
